# Fruit Bag Removal Timing Influences Fruit Coloration, Quality, and Physiological Disorders in ‘Arisoo’ Apples

**DOI:** 10.3390/plants14182923

**Published:** 2025-09-20

**Authors:** Nay Myo Win, Van Giap Do, Jung-Geun Kwon, Jong-Taek Park, Juhyeon Park, Youngsuk Lee, Hun-Joong Kweon, In-Kyu Kang, Soon-Il Kwon, Seonae Kim

**Affiliations:** 1 Apple Research Center, National Institute of Horticultural and Herbal Science, RDA, Daegu 43100, Republic of Korea; naymyowin@korea.kr (N.M.W.); giapbio@korea.kr (V.G.D.); dsms0@korea.kr (J.-G.K.); jongtaek@korea.kr (J.-T.P.); wngus1113@korea.kr (J.P.); kongfo@korea.kr (Y.L.); kweonhj@korea.kr (H.-J.K.); topapple@korea.kr (S.-I.K.); 2 Department of Horticultural Science, Kyungpook National University, Daegu 41566, Republic of Korea; kangik@knu.ac.kr

**Keywords:** bagging, fruit quality, physiological disorders, anthocyanin, carotenoid, chlorophyll

## Abstract

The timing of fruit bag removal is important for achieving optimum fruit quality, coloration, and visual appearance. Therefore, this study investigated the effects of fruit bag removal timing on fruit quality and color and the occurrence of physiological disorders in ‘Arisoo’ apples. Fruits were bagged in two-layer paper bags, which were removed 30, 20, and 10 days before harvest (DBH). Unbagged fruits served as the control. The incidence of fruit cracking, sunburn, and pathogen infection was highest in the unbagged group, followed by the group with bag removal at 30 DBH, and lowest in those with bag removal at 20 and 10 DBH. However, bag removal at 10 DBH significantly reduced fruit weight and soluble solids content but increased firmness. Additionally, bag removal at 10 DBH resulted in poorly colored fruits with higher chlorophyll and lower anthocyanin and carotenoid pigments and lower expression levels of pigment-related genes, including anthocyanin-, carotenoid-, and chlorophyll degradation-associated genes, compared with those in the other treatment groups. Bag removal at 30 and 20 DBH did not significantly affect fruit quality or coloration, but it did affect fruit size. Overall, this study serves as a reference for determining the optimal timing of fruit bag removal to enhance the quality and coloration of ‘Arisoo’ apples.

## 1. Introduction

Apples (*Malus domestica*) are economically important crops; therefore, high-quality apples with better coloration and appearance enhance profitability [1]. ‘Arisoo’ is a widely cultivated and mid-season apple cultivar, valued for its attractive color, flavor, and quality, with an estimated harvest period in early September [2]. In apple production, adequate sunlight exposure and temperature are required not only to improve photosynthesis but also to promote fruit quality and skin color pigment development [3]. However, the increase in daily temperatures and solar radiation due to global warming and climate change significantly affects fruit crop production, including that of apples [4,5]. In Korea, the frequency of heat waves and elevated temperatures in summer (from June to August) has noticeably increased over the past decades, leading to an increased risk of physiological disorders (including sunburn and cracking) in fruit crops [6], affecting the quality of apples [7]. Generally, heat stress induces oxidative damage and physiological disorders, resulting in low-quality fruits [8,9,10]. Fruit cracking and pathogen infection are highly associated with environmental factors, including temperature and frequent rainfall conditions [11]. Therefore, fruit damage to apples caused by high temperatures and other environmental conditions is expected to increase in the future, necessitating protective methods to alleviate fruit damage and produce high-quality fruits.

Fruit bagging is an agricultural strategy that is widely used to protect fruits from damage caused by high-temperature and rainfall conditions [12]. Fruit bagging can extensively improve the color and appearance of apples, with bagged fruits having smoother, cleaner, and shinier surfaces and being free from sunburn and pathogen infection [13,14,15]. Light is an important factor for inducing fruit color pigments, with anthocyanins and carotenoids accumulating on the skin of apples, peaches (*Prunus persica*), and kiwifruits (*Actinidia* spp.) after bag removal [16,17,18]. However, a longer bagging duration and late removal close to harvesting can negatively affect fruit size, quality, nutrient content, and skin color pigments [12,19,20]. Additionally, the non-removal of fruit bags results lower anthocyanin and higher chlorophyll contents [16]. Therefore, the timing of fruit bag removal should be carefully considered not only to reduce physiological disorders but also to improve apple fruit color and quality.

The pigments in apple skin intensify during fruit maturation due to anthocyanin and carotenoid accumulation [21] and chlorophyll degradation [22]. Several key genes are involved in the improvement of fruit color pigments. Generally, *MdPAL*, *MdCHS*, *MdCHI*, *MdF3H*, *MdDFR*, *MdANS*, and *MdUFGT* genes and *MdGGPPS*, *MdPSY*, *MdZISO*, *MdPDS*, *MdZDS*, *MdCRTISO*, *MdLCYε*, *MdLCYβ*, *MdCRHβ*, and *MdZEP* genes are considered major genes in the anthocyanin and carotenoid biosynthesis pathways, respectively, and their expressions are closely related to anthocyanin and carotenoid accumulation in apples [21,23,24]. In addition, transcription factor genes (*MdMYB10*, *MdMYB1*, and *MdMYB110a*) promote anthocyanin biosynthesis [23,25]. In contrast, the expression of chlorophyll degradation-associated genes (*MdNYC1*, *MdNYC3*, *MdNOL2*, *MdHACR*, *MdNYE1a*, *MdPPH1*, *MdPAO6*, and *MdRCCR2*) promotes chlorophyll breakdown, and the loss of green coloration during fruit maturation is related to the expression of these genes [22,26]. Additionally, the degreening of apple fruits is related to the development of red coloration [27]. In general, bagging hinders light availability to fruits, and anthocyanin and carotenoid accumulation is reactivated upon bag removal and fruits are re-exposed to sunlight, leading to the upregulation of anthocyanin and carotenoid biosynthesis-associated genes [28,29]. However, reports on how fruit bag removal timing affects apple quality and coloration, and how late fruit bag removal timing results in poorly colored fruits are limited. Therefore, considering the optimal fruit bag removal timing to achieve greater marketability is essential.

Therefore, this study aimed to evaluate the effects of fruit bag removal timing on fruit quality and the incidence of physiological disorders of ‘Arisoo’ apples at commercial harvest. Additionally, anthocyanin and carotenoid accumulation, chlorophyll degradation, and their related gene expression levels at different bag removal times were investigated.

## 2. Results

### 2.1. Incidence of Fruit Cracking, Sunburn, and Pathogen Infection

**Figure 1:**
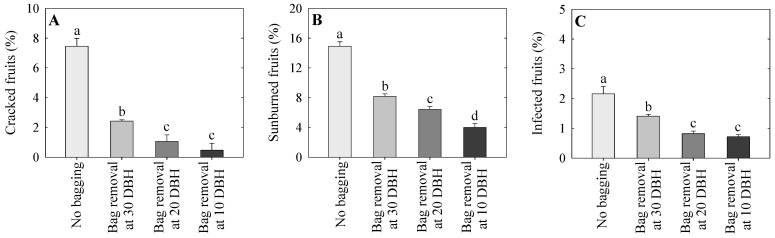
The incidence rates of cracking (**A**), sunburn (**B**), and infection (**C**) in ‘Arisoo’ apple trees following control (no bagging) and bag removal at 30, 20, and 10 days before harvest (DBH), as observed at harvest. The data indicate mean ± standard error; lowercase letters indicate significant differences based on Tukey’s HSD test at *p* < 0.05.

The incidence rates of cracked, sunburned, and infected fruits were significantly higher in the non-bagged group than those in the bagged treatment groups (Figure 1A–C). The incidence rates of fruit cracking were 67.5, 85.8, and 93.8% higher in the non-bagged group than those in the groups with bag removal at 30, 20, and 10 days before harvest (DBH), respectively (Figure 1A). Additionally, the fruit cracking incidence rates were 13.4 and 19.3% higher in the group with bag removal at 30 DBH than those in the groups with bag removal at 20 and 10 DBH, respectively. Although the fruit cracking rate in the group with bag removal at 20 DBH was slightly higher (8.0%) than that in the group with bag removal at 10 DBH, the difference did not reach statistical significance (Figure 1A).

Sunburn incidence was 45.2, 57.1, and 73.3% higher in the non-bagged group than in the 30, 20, and 10 DBH bag removal groups, respectively (Figure 1B). The sunburn incidence rates were 11.9 and 28.1% higher in the 30 DBH bag removal group than in the 20 and 10 bag removal groups, respectively. Additionally, sunburn incidence was 16.2% higher in the 20 DBH bag removal group than that in the 10 DBH bag removal group (Figure 1B).

The incidence rates of infected fruits were 34.8, 62.2, and 66.6% higher in the non-bagged group than those in the 30, 20, and 10 DBH bag removal groups, respectively (Figure 1C). Additionally, the incidence rates of infected fruits were 27.5 and 31.9% higher in the 30 DBH bag removal group than those in 20 and 10 DBH bag removal groups, respectively. The incidence rate of infected fruits did not differ between the 20 and 10 DBH bag removal groups (Figure 1C).

### 2.2. Fruit Surface Temperature, Fruit Weight, and Size

The fruit surface temperature between non-bagged and bagged fruits was measured (Figure S1). The fruit surface temperature of non-bagged fruits was 40.0 °C, while that of bagged fruits was 36.2 °C (Figure S1).

Fruit weight and size were significantly affected by bagging (Table 1). Fruit weight significantly increased in the non-bagged group, whereas bag removal near harvest (20 and 10 DBH bag removal groups) produced small-sized fruits. Fruit weight between the non-bagged and 30 DBH bag removal groups was not significantly different. Additionally, the 30 DBH bag removal group produced heavier fruit than the 20 and 10 DBH bag removal groups. Fruit size (length and diameter) was larger in the non-bagged group than in the 10 DBH bag removal group. However, the shape (length: diameter ratio) of the fruit did not differ among the treatment groups (Table 1).

### 2.3. Fruit Quality and Color

Regarding fruit quality characteristics, fruits from the 10 DBH bag removal group exhibited higher flesh firmness compared with that in the non-bagged group (Table 2). However, flesh firmness did not significantly differ between the 20 and 10 DBH bag removal groups. The non-bagged group had a higher soluble solids content (SSC) than that of the 10 DBH bag removal. The SSC of the 30 and 20 DBH bag removal groups was not significantly different from those of the non-bagged and 10 DBH bag removal groups. However, no significant differences were observed in titratable acidity (TA) and starch pattern index values among the treatment groups. The SSC:TA ratio was higher in the non-bagged group than in the 10 DBH bag removal group.

Fruit bagging significantly affected the development of fruit skin coloration (Table 2). The L* value was highest in the 10 DBH bag removal group and lowest in the non-bagged group. Additionally, the L* value of the 30 DBH bag removal and non-bagged groups did not differ at harvest. The highest *C* value was observed in the 10 DBH bag removal treatment compared with that in the treatment groups. The *h*^o^ value was highest in the 10 DBH bag removal group, followed by the 20 and 30 DBH bag removal group, and lowest in the non-bagged group. The fruit color indicators (L*, *C*, and *h*^o^) did not significantly differ between the 30 and 20 DBH bag removal groups.

**Figure 2:**
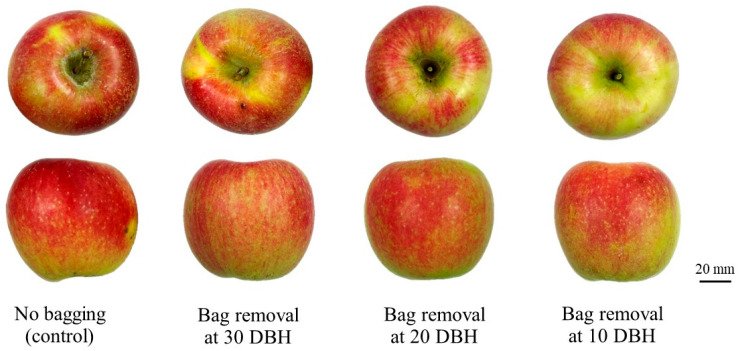
Representative phenotypic images of ‘Arisoo’ apples following control (no bagging) and bag removal at 30, 20, and 10 days before harvest (DBH), as observed at harvest.

Figure 2 presents fruit sample images of ‘Arisoo’ apples at harvest, collected from the non-bagged, 30, 20, and 10 DBH bag removal groups.

### 2.4. Chlorophyll, Anthocyanin, and Carotenoid Contents

**Figure 3:**
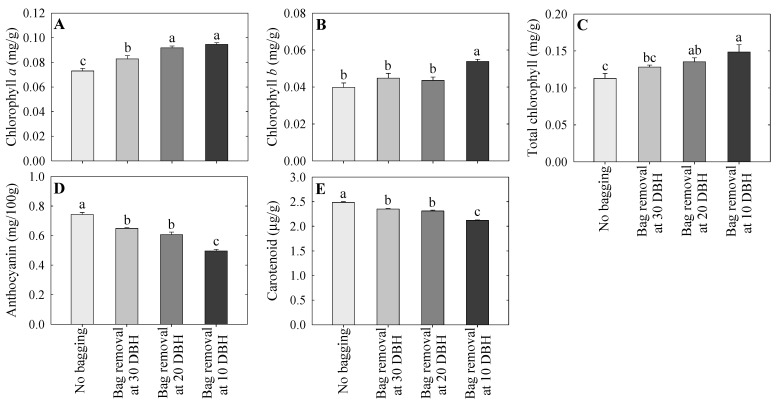
Chlorophyll *a* (**A**), chlorophyll *b* (**B**), and total chlorophyll (**C**), anthocyanin (**D**), and carotenoid (**E**) contents in the fruit skins of ‘Arisoo’ apples following control (no bagging) and bag removal at 30, 20, and 10 days before harvest (DBH), as observed at harvest. The data indicate mean ± standard error; lowercase letters indicate significant differences based on Tukey’s HSD test at *p* < 0.05.

In the present study, fruit color pigments (chlorophyll *a*, chlorophyll *b*, and total chlorophyll, anthocyanins, and carotenoids,) were analyzed, as shown in Figure 3. The chlorophyll *a* content was highest in the 10 and 20 DBH groups, followed by the 30 DBH group, and lowest in the non-bagged group (Figure 3A). The highest chlorophyll *b* was observed in the 10 DBH bag removal treatment compared with that in the treatment groups (Figure 3B). The total chlorophyll content was highest in the 10 DBH bag removal group and lowest in the non-bagged group (Figure 3C). The total chlorophyll content in the 20 DBH bag removal group was not significantly different from that in the 10 or 30 DBH groups. Similar total chlorophyll contents were observed in the non-bagged and 30 DBH groups. Unlike total chlorophyll, anthocyanin content (cyanidin-*3*-galactosidase) was highest in the non-bagged group, followed by the 30 and 20 DBH groups, and lowest in the 10 DBH group (Figure 3D). Similar to anthocyanins, carotenoid content (β-carotene) was highest in the non-bagged group, followed by the 30 and 20 DBH groups, and lowest in the 10 DBH group (Figure 3E).

### 2.5. Expression Analysis of Chlorophyll Degradation-Associated Genes

**Figure 4:**
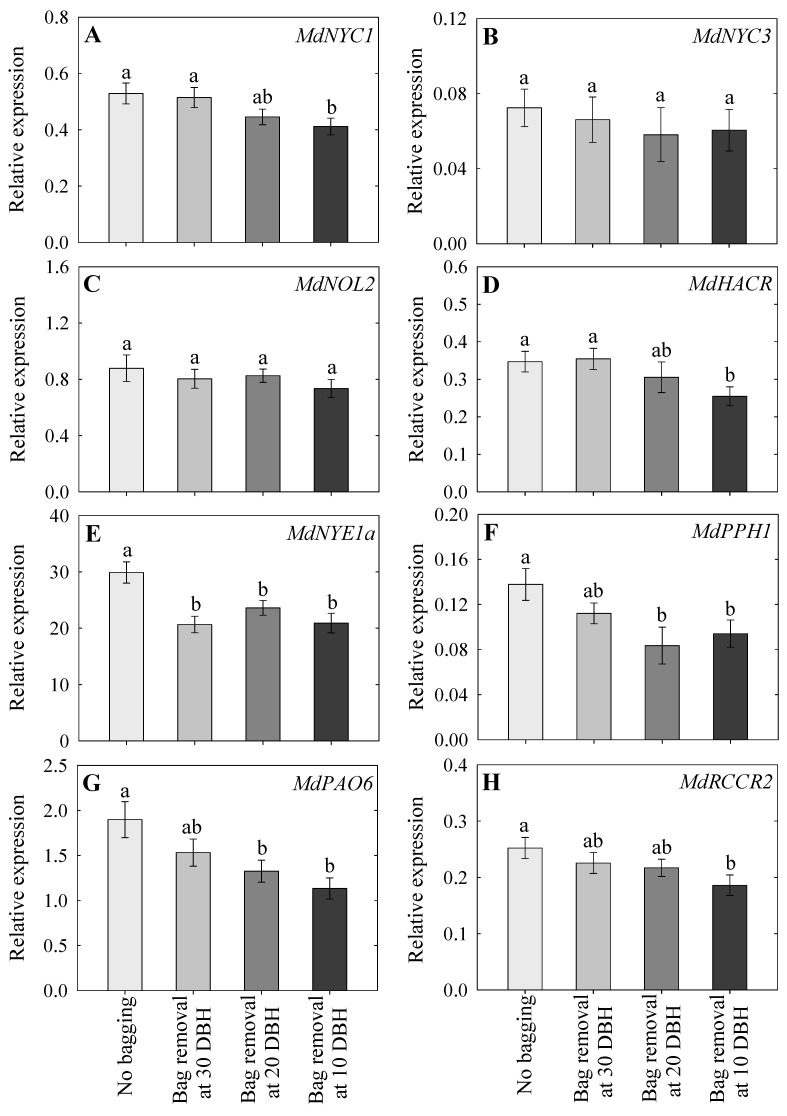
Relative expression levels of chlorophyll degradation-associated genes, including *MdNYC1* (**A**), *MdNYC3* (**B**), *MdNOL2* (**C**), *MdHACR* (**D**), *MdNYE1a* (**E**), *MdPPH1* (**F**), *MdPAO6* (**G**), and *MdRCCR2* (**H**), following no bagging (control) and bag removal at 30, 20, and 10 days before harvest (DBH), as observed at harvest. The data indicate mean ± standard error; lowercase letters indicate significant differences based on Tukey’s HSD test at *p* < 0.05.

The expression levels of chlorophyll degradation-associated genes (*MdNYC1*, *MdHACR*, *MdNYE1a*, *MdPPH1*, *MdPAO6*, and *MdRCCR2*) in the non-bagged control fruits were significantly higher than those in the 10 DBH bag removal group, except for *MdNYC3* and *MdNOL2* (Figure 4A–H). *MdNYE1a*, *MdPPH1*, and *MdPAO6* expression in the non-bagged group was also higher than that in the 20 DBH group (Figure 4E–G). Except for *MdNYE1a*, the expression of all chlorophyll degradation-associated genes did not differ between the 30 DBH and non-bagged groups (Figure 4A–H). Additionally, the expression of all chlorophyll degradation-associated genes did not differ between the 30 and 20 DBH bag removal groups (Figure 4A–H).

### 2.6. Expression Analysis of Anthocyanin-Associated Genes

**Figure 5:**
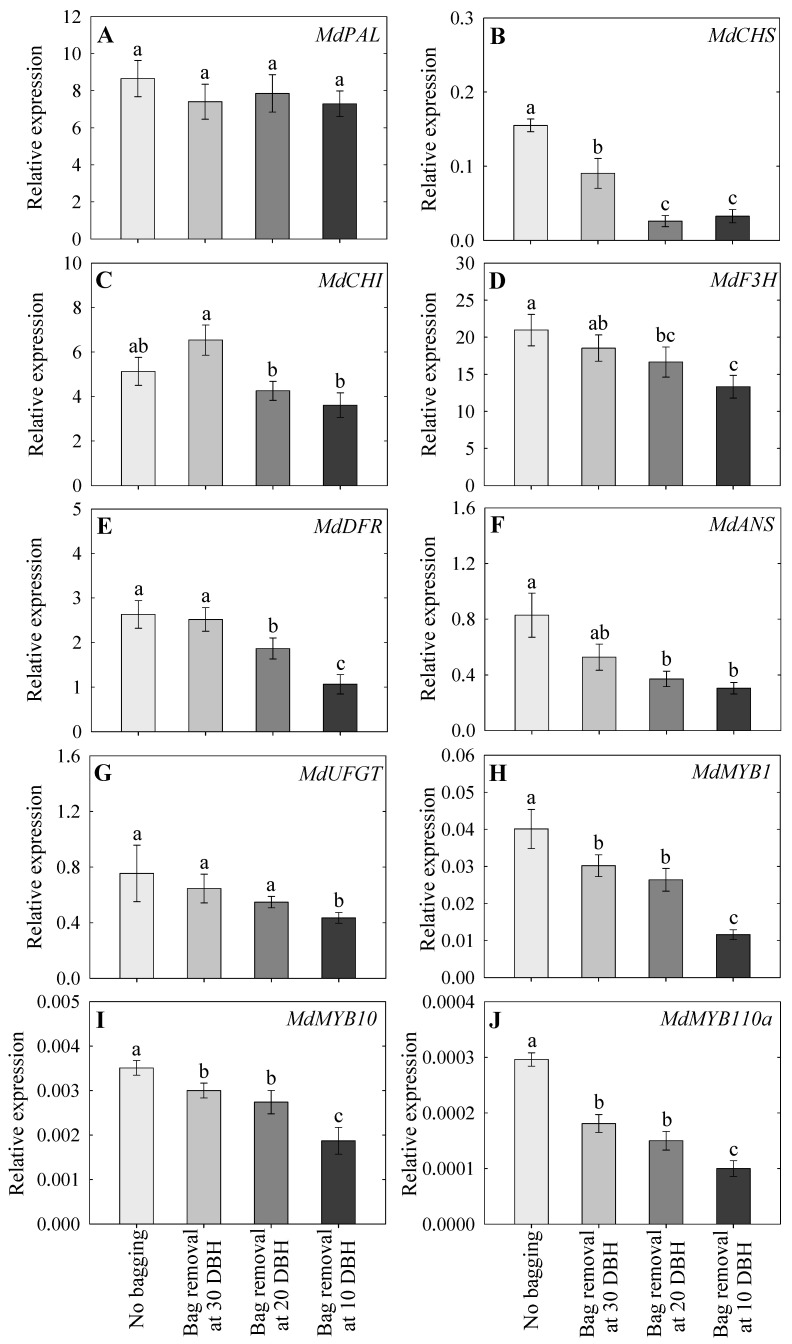
Relative expression levels of anthocyanin genes, including *MdPAL* (**A**), *MdCHS* (**B**), *MdCHI* (**C**), *MdF3H* (**D**), *MdDFR* (**E**), *MdANS* (**F**), *MdUFGT* (**G**), *MdMYB1* (**H**), *MdMYB10* (**I**), and *MdMYB110a* (**J**), following non-bagging (control) and bag removal at 30, 20, and 10 days before harvest (DBH), as observed at harvest. The data indicate mean ± standard error; lowercase letters indicate significant differences based on Tukey’s HSD test at *p* < 0.05.

The expression levels of anthocyanin biosynthesis (*MdPAL*, *MdCHS*, *MdCHI*, *MdF3H*, *MdDFR*, *MdANS*, and *MdUFGT*) and transcription factor (*MdMYB1*, *MdMYB10*, and *MdMYB110a*) genes were analyzed (Figure 5A–J). The expression of anthocyanin biosynthesis genes was upregulated in all treatments, with the highest expression observed in the non-bagged group and the lowest in the 10 DBH bag removal group. However, *MdPAL* and *MdCHI* were not significantly different between the non-bagged and 10 DBH groups (Figure 5A,C). Except for *MdPAL* and *MdANS*, all anthocyanin gene expression levels were higher in the 30 DBH bag removal group than those in the 10 DBH group (Figure 5A–J). Additionally, the expression levels of *MdDFR*, *MdUFGT*, *MdMYB1*, *MdMYB10*, and *MdMYB110a* were higher in the 20 DBH bag removal group than those in the 10 DBH group (Figure 5E,G–J). Compared with those in the non-bagged group, bag removal at 30 DBH resulted in lower *MdCHS*, *MdMYB1*, *MdMYB10*, and *MdMYB110a* expression levels (Figure 5B,H–J). Additionally, bag removal at 30 DBH resulted in higher *MdCHS*, *MdCHI*, and *MdDFR* expression levels than those in the 20 DBH bag removal group; however, the expression levels of the other genes did not significantly differ between the two treatment groups (Figure 5A–J).

### 2.7. Expression Analysis of Carotenoid-Associated Genes

**Figure 6:**
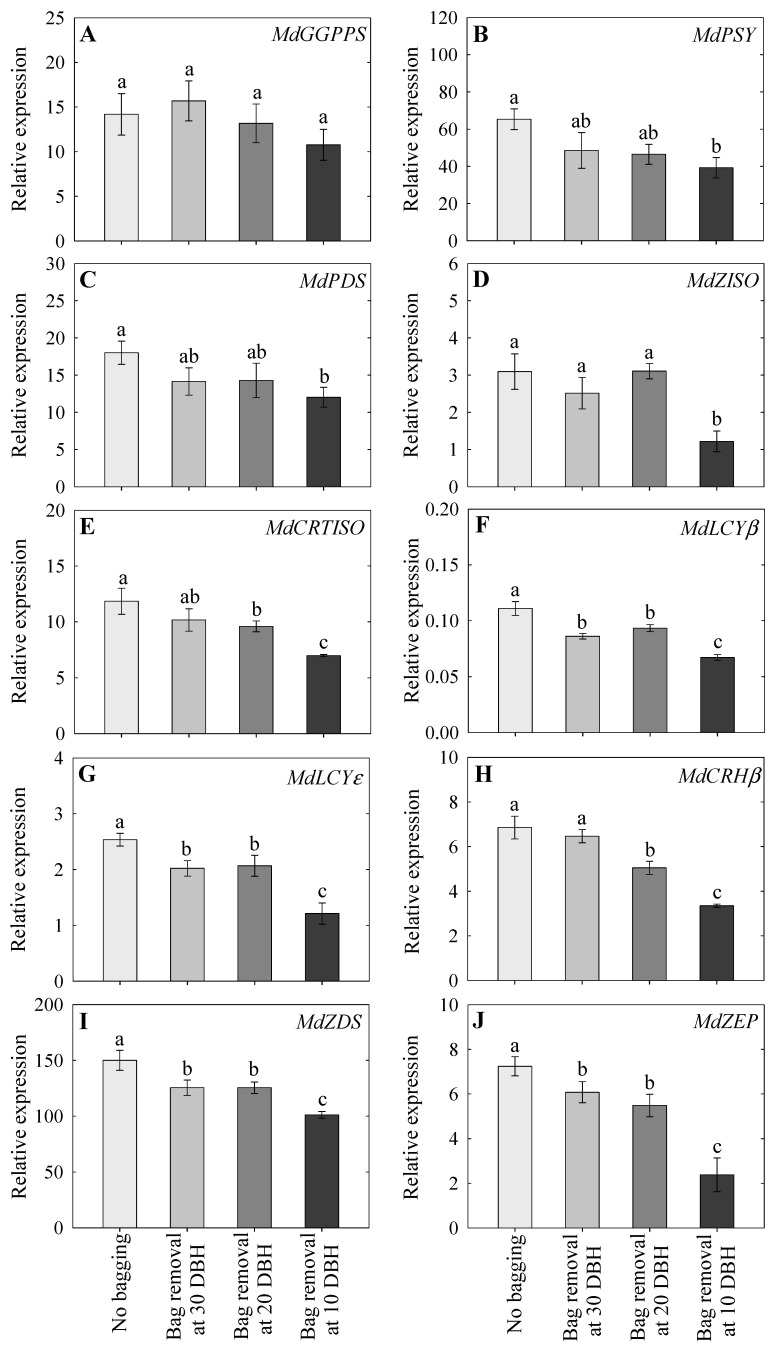
Relative expression levels of carotenoid genes, including *MdGGPPS* (**A**), *MdPSY* (**B**), *MdPDS* (**C**), *MdZISO* (**D**), *MdCRTISO* (**E**), *MdLCYb* (**F**), *MdLCYe* (**G**), *MdCRHb* (**H**), *MdZDS* (**I**), and *MdZEP* (**J**), following non-bagging (control) and bag removal at 30, 20, and 10 days before harvest (DBH), as observed at harvest. The data indicate mean ± standard error; lowercase letters indicate significant differences based on Tukey’s HSD test at *p* < 0.05.

The expression levels of carotenoid-associated genes (*MdGGPPS*, *MdPSY*, *MdZISO*, *MdPDS*, *MdZDS*, *MdCRTISO*, *MdLCYε*, *MdLCYβ*, *MdCRHβ*, and *MdZEP*) were also analyzed (Figure 6). Carotenoid gene expression was upregulated in all treatments, with the highest expression observed in the non-bagged group and the lowest in the 10 DBH bag removal group. Except for *MdGGPPS*, the expression of all carotenoid genes was significantly higher in the non-bagged group than in the 10 DBH group (Figure 6A–J). Higher carotenoid gene expression levels were also observed in the 30 and 20 DBH bag removal groups than those in the 10 DBH group, except for *MdGGPPS*, *MdPSY*, and *MdPDS* expression (Figure 6A–J). Compared with those in the non-bagged group, the 30 DBH bag removal group exhibited lower *MdLCYβ*, *MdLCYε*, *MdZDS*, and *MdZEP* expression levels (Figure 6A–J). However, aside from the lower expression levels of *MdCRHβ*, the expression levels of other carotenoid genes were not significantly different between the 30 and 20 DBH bag removal groups (Figure 6A–J).

### 2.8. PCA Score and Loading Plots

**Figure 7:**
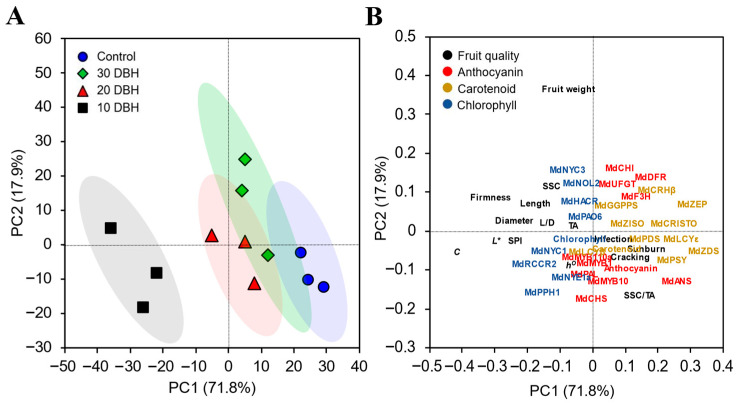
Principal component analysis (PCA) score (**A**) and loading (**B**) plots for ‘Arisoo’ apples with non-bagging (control) and removal of fruit bags at 30, 20, and 10 days before harvest (DBH).

The PCA score and loading plots were used to evaluate the overall variation in data responses resulting from different fruit bag removal timing (Figure 7). The variances explained by the PCA score and loading plots were 71.8 and 17.9% of PC-1 and PC-2, respectively. The PCA score plot revealed separation between treatments, with the 10 DBH bag removal group showing the greatest dispersion, whereas the 30 DBH bag removal group showed the least dispersion relative to the non-bagged group (Figure 7A). Bag removal at 20 and 30 DBH resulted in closely clustered dispersion patterns. Furthermore, the PCA loading plot showed a strong relationship between sunburn, cracking, and infection, indicating that physiological disorders were likely co-regulated (Figure 7B). The strong association among transcription factor genes, anthocyanins, and *h*^o^ value suggests that transcription factors may be induced by red pigments. Additionally, carotenoid-associated genes were observed in the same region, indicating that these genes are closely regulated by carotenoid accumulation (Figure 7B).

## 3. Discussion

In the present study, improvements in fruit quality and coloration, as well as the reduction in physiological disorders, varied depending on the timing of fruit bag removal. Fruit cracking is the physical failure of the skin that presents as a fracture of the cuticle or epidermal layers under stressful conditions [30]. Generally, physiological, environmental, and genetic variations are the primary factors that cause fruit cracking [31]. Exposure of the fruit surface to water at high temperatures and humidity can induce the formation of microcracks in the cuticle, leading to cracking [32]. Fruit bagging can protect fruits from various abiotic and biotic stresses [12]. Thus, the use of rainproof cultivation and bagging reduces fruit cracking [33]. Additionally, fruit bagging reduces the incidence of internal ring cracking and stem-end splitting in ‘Fuji’ apples [34]. Consistent with previous studies, bagging significantly reduced fruit cracking in all bagging treatments, particularly in the 10 DBH bag removal group.

Fruit sunburn, a physiological disorder caused by excessive sunlight radiation, generally occurs in fruit crops growing in warm climates [35]. Fruit surfaces exposed to prolonged sunlight or high temperatures for a period of time exhibit sunburn [36]. Fruit bagging prevents sunburn in apples by reducing the fruit surface temperature and minimizing heat stress [37]. In the current study, the fruit surface temperature in the bagged fruits was lower than that of non-bagged fruits. Additionally, the highest sunburn incidence was observed in non-bagged fruits, whereas bag removal at 10 DBH reduced sunburn incidence. Therefore, the reduction in fruit sunburn rate in the bagged fruits may be attributed to lower fruit surface temperature and consequently reduced heat stress in fruits. These findings indicated that apples without protective (non-bagged) treatments displayed increased fruit sunburn, whereas the risks of sunburn were substantially reduced by bagging under high-temperature conditions.

Fruit bagging is an effective way to control pests and diseases in fruits [15]. Two-layer bags effectively reduce pathogen infections in apples [13]. Sharma et al. [38] also reported that fruit bagging noticeably reduced the incidence of bitter pits in apples. Importantly, fruit bagging reduces pesticide residues on apple surfaces, thereby lowering chemical residue levels in fresh fruit consumption [14]. In the present study, the fruit infection rate was significantly reduced in the late removal of fruit bags, whereas non-bagged fruits exhibited increased pathogen infection. Moreover, the PCA loading plot showed that physiological disorders were likely to co-occur, and fruit bagging remarkably mitigated the risks of these disorders.

Fruit bagging also influences apple quality. The 10 DBH bag removal group showed higher firmness and a lower SSC than those in the non-bagged group. Additionally, the SSC:TA ratio was highest in the non-bagged group and lowest in the 10 DBH bag removal group. Fruit weight was noticeably reduced in the 10 and 20 DBH bag removal groups, whereas the non-bagged group exhibited the largest fruit size. Ali et al. [12] reported that a longer fruit bagging duration can lead to the production of smaller fruits. Fruit bagging negatively affects the fruit size of pomegranate [39] and results in small-sized apples [40], as observed in this study. Therefore, delayed fruit bag removal near harvest time was assumed to result in the production of smaller fruits with low sugar levels. Variations in fruit size and sugar content also depend on bag application timing, type, and color, cultivar, and environmental conditions [15].

Do et al. [21] reported that anthocyanins and carotenoids develop in apple skin and gradually increase as the fruits mature. Additionally, the increase in fruit color pigments is attributed to the expression of anthocyanin- and carotenoid-associated genes, which are induced by sunlight [41]. Win et al. [42] reported that improved sunlight exposure in apples during fruit maturation significantly enhances anthocyanin pigments. In peaches, the development of fruit peel color is influenced by bagging duration, with early bag removal to promote sunlight exposure improving red coloration and anthocyanin levels [17]. Ma et al. [29] reported that anthocyanin levels steadily increased in the ‘Granny Smith’ apples after bag removal. In the present study, the non-bagged fruits had the highest anthocyanin and carotenoid contents, whereas the late removal of fruit bags (particularly at 10 DBH) resulted in the lowest pigment content. Fruit skin color indicators revealed that the non-bagged group had the lowest *h*^o^ value, whereas the 10 DBH bag removal group exhibited the highest *h*^o^ value. The PCA score plot also revealed spatial segregation between the early bag removal (10 DBH) and non-bagged groups. Therefore, our results indicate that the timing of bag removal is crucial for achieving sufficient light exposure duration to induce pigment accumulation in apples, and that late bag removal could lead to low fruit coloration.

In the current study, the chlorophyll content was lowest in the non-bagged fruit, followed by the 30 and 20 DBH bag removal groups, and highest in the 10 DBH bag removal group. Generally, the chlorophyll degradation process is regulated by multi-enzyme activities [22]. Therefore, the key genes that mainly involved in the chlorophyll degradation pathway (*MdNYC1*, *MdNYC3*, *MdNOL2*, *MdHACR*, *MdNYE1a*, *MdPPH1*, *MdPAO6*, and *MdRCCR2*) were selected and their expression levels were measured in this study. Additionally, chlorophyll degradation-associated gene expression levels were highest in the non-bagged group and lowest in the 10 DBH group. Lv et al. [22] reported that pheophorbide a oxygenase (PAO) and red chlorophyll catabolite reductase (RCCR) are responsible for the loss of green coloration during chlorophyll degradation. Chlorophyll breakdown is a key process during fruit maturation, and increased pheophytinase (PPH), PAO, and RCCR gene expression has been observed throughout this process [26], consistent with the present study. Additionally, the development of red coloration is related to chlorophyll breakdown in ‘Red Fuji’ apples [27]. However, bagging can alter chlorophyll content, with extended fruit bagging resulting in a higher chlorophyll content [43]. Thus, the current study indicated that chlorophyll was highly degraded in the non-bagged fruits, but remained high in the 10 DBH bag removal group, which might be due to the delayed removal of fruit bags.

The process of anthocyanin accumulation is regulated by the activities of anthocyanin biosynthesis enzymes [21]. Therefore, the key genes that mainly involved in the anthocyanin biosynthesis pathway (*MdPAL*, *MdCHS*, *MdCHI*, *MdF3H*, *MdDFR*, *MdANS*, and *MdUFGT*) were selected and their expression levels were measured in this study. The expression of anthocyanin biosynthesis and transcription factor genes increased in all treatments, with the highest expression observed in the non-bagged group, followed by 30, 20, and 10 DBH bag removal groups. Generally, anthocyanin synthesis is controlled by structural genes that are highly expressed during the fruit maturation stage [21,44]. Espley et al. [25] reported that transcription factor genes, especially *MdMYB10*, regulate the development of red coloration in apples, and their expression is closely related to anthocyanin accumulation. Similarly, Gao et al. [24] reported that *MdMYB10* and *MdMYB110a* are positively associated with anthocyanin biosynthesis, and *MdMYB1* activates the expression of anthocyanin biosynthesis genes to promote anthocyanin accumulation. Wang et al. [28] argued that the expression of anthocyanin biosynthesis genes was upregulated after bag removal. Additionally, Feng et al. [16] found that anthocyanin progressively accumulated, accompanied by increased expression of transcription factors and structural genes, as the duration of bag removal increased. Therefore, the accumulation of red pigments is strongly associated with the expression of anthocyanin biosynthesis genes, which are regulated by transcription factor genes, as observed in the present study.

The process of carotenoid accumulation is regulated by the activities of carotenoid biosynthesis enzymes [21]. Therefore, the key genes that mainly involved in the carotenoid biosynthesis pathway (*MdGGPPS*, *MdPSY*, *MdZISO*, *MdPDS*, *MdZDS*, *MdCRTISO*, *MdLCYε*, *MdLCYβ*, *MdCRHβ*, and *MdZEP*) were selected and their expression levels were measured in this study. Similar to anthocyanins, the expression of carotenoid genes increased in all treatments, with the highest expression observed in non-bagged fruits, followed by 30 and 20 DBH bag removal groups, and lowest in the 10 DBH bag removal group. The PCA loading plot also indicated a close relationship between carotenoid genes, suggesting that carotenoid accumulation was regulated by the coordination of these genes. Do et al. [21] reported that carotenoid gene expression progressively increases when fruits mature. Increased carotenoid accumulation and carotenoid gene expression at the fruit maturity stage have been reported in apples [45]. Light induced carotenoid accumulation in apple and bagged fruits results in lower carotenoid content and carotenoid gene expression levels [46]. Therefore, the lower carotenoid content and lower expression patterns observed in the 10 DBH bag removal group might be due to the late removal of fruit bags near commercial harvest time. As previously mentioned, sufficient duration of exposure to sunlight after bag removal is required for pigment accumulation.

Overall, different bag removal timing resulted in a broad range of apple qualities. The non-bagged treatment exhibited high anthocyanin and carotenoid contents but increased physiological disorders in fruits. Fruit bagging can prevent fruit damaging and improve both the quality and visual appearance of apples. However, late bag removal at 10 DBH led to low anthocyanin and carotenoid contents, although it reduced physiological disorders in fruits. Therefore, the bag removal at 20 and 30 DBH appears to be more beneficial not only to improve fruit quality and color but also reduce fruit damage. Moreover, the potential variability of the results and continuous studies of fruit bagging techniques should be considered to ensure the efficient production of high-quality fruits across different growing seasons.

## 4. Materials and Methods

### 4.1. Experimental Orchard and Tree Selection

**Figure 8:**
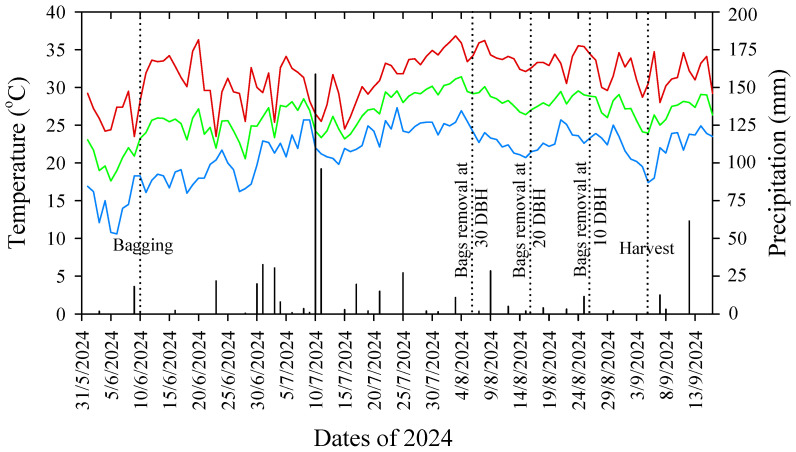
Daily temperature and precipitation in 2024 in the experimental orchard located in Yeongcheon-si, Gyeongsangbuk-do Province, Korea. Fruits were bagged on 10 June 2024, and fruit bags were removed at 30 (6 August 2024), 20 (16 August 2024), and 10 (26 August 2024) days before harvest (DBH). All treatment fruits were harvested on 5 September 2024. The red, blue, and yellow color lines indicate the maximum, minimum, and average temperatures, respectively, and black-colored bars indicate precipitation in the experimental orchard.

The experiment was conducted in a commercial apple orchard located in Yeongcheon-si, Gyeongsangbuk-do Province, South Korea. Five-year-old ‘Arisoo’ apple trees with a uniform canopy size and planted in the same soil and environmental conditions at a 3.0 m × 1.0 m spacing were selected for this study. The trees were grafted onto M.9 rootstocks and irrigated using sprinkler and drip irrigation systems. The experimental field was managed using an integrated pest management system. Flowers fully bloomed in the orchard on 22 April 2024, and the crop load levels of the trees were adjusted to eight fruits per cm^2^ of trunk cross-sectional area after 3 weeks of full bloom. The daily temperature and precipitation in the experimental orchards are presented in Figure 8.

### 4.2. Fruit Bagging Treatments

**Figure 9:**
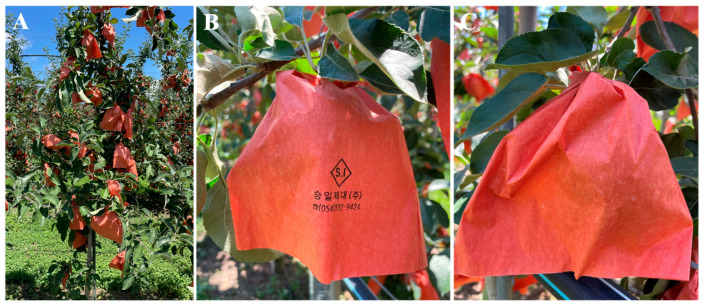
The ‘Arisoo’ apple trees used in this study (**A**). Front (**B**) and back (**C**) views of bagged apple fruits. Fruits were bagged on 10 June 2024.

Four fruit-bagging treatment groups were used. Thirty-six apple trees were used in the four treatments, with each treatment comprising nine apple trees with three replicates (three per replicate). The first group was designated as the control group, containing unbagged fruits (non-bagged). All fruits from the second to fourth treatment groups were bagged using two-layer paper bags, 18 cm long and 15 cm wide, which were specially designed for apple bagging (Seungiljedae Co., Yeongcheong, Republic of Korea). Fruit bagging was performed on 10 June 2024. Figure 9 shows the apple trees after bag treatment, along with the front and back views of the bagged apples. Fruit bags from all bagged treatment groups were subsequently removed at different times: 30 days before harvest (DBH) (6 August 2024), 20 DBH (16 August 2024), and 10 DBH (26 August 2024). All fruits were harvested on 5 September 2024.

### 4.3. Determination of Fruit Cracking, Sunburn, and Infection Incidence

To determine the effects of fruit-bagging on fruit cracking, sunburn incidence, and pathogen infection, individual fruits from each apple tree were visually inspected and counted before fruit-bagging and at harvest. The incidence of cracked fruit was calculated using the equation described by Wang et al. [47]. The incidence of sunburned fruits (all types of sunburned fruits) was calculated using the equation of Yoo et al. [48]. The infected fruit rate (all types of infection) was calculated using the equation described by Do et al. [49].

Fruit cracking incidence rate (%) = (number of cracked fruits per plant/total number of fruits per plant) × 100.

Sunburned fruit incidence rate (%) = (number of sunburned fruits per plant/total number of fruits per plant) × 100.

The infected fruit rate (%) = (number of infected fruits per plant/total number of fruits per plant) × 100.

### 4.4. Determination of Fruit Surface Temperature and Fruit Quality Characteristics

The fruit surface temperature of non-bagged and bagged fruits was measured at midday on a sunny day (1 August 2024) using an IR camera (FLIR E50, Oriontek Co., Gyeonggi, Republic of Korea). Fruit size (length and diameter) was measured using a digital caliper, and fruit weight was measured using a digital scale. The fruit color values (L*, a*, and b*) of the apple skin were measured using a chroma meter (CR-400; Konica Minolta, Tokyo, Japan), and the results were converted to chroma (*C*) and hue angle (*h*^o^) values using the formula described by Giap et al. [41]. Fruit firmness was measured on three random locations on each fruit using a fruit texture analyzer equipped with an 11 mm plunger (TR Turoni, Forlì, Italy). The soluble solids content (SSC) of fruit was determined in a fruit juice sample using a refractometer (PR-201α; Atago, Tokyo, Japan). The titratable acidity (TA) of the fruit was measured following a malic acid reduction method using 0.1 N NaOH, as described by Win et al. [42]. During fruit quality assessment, fruit peel samples were collected and stored for further pigment analysis.

### 4.5. Determination of Chlorophyll, Anthocyanin, and Carotenoid Contents

The chlorophyll contents (chlorophyll *a*, chlorophyll *b*, and total chlorophyll) was determined following the method described by Lichtenthaler [50,51]. Peel tissue samples were extracted with 80% acetone. The extracts were then centrifuged, and the supernatant was collected. The absorbance of the collected supernatants was determined at 645 and 662 nm, and the chlorophyll contents were calculated as follows: Chlorophyll *a* = (11.24 × A_662_) − (2.04 × A_645_); Chlorophyll *b* = (20.13 × A_645_) − (4.19 × A_662_); Total chlorophyll = (7.05 × A_662_) + (18.09 × A_645_).

Anthocyanin content was determined using the pH differential method [21]. The peel samples were homogenized with 80% acetone and then filtered through a filter paper. Next, the filtrates were evaporated using a rotary evaporator, and the volume of the sample was adjusted to 10 mL with distilled water. The samples were mixed potassium chloride buffer (pH_1.0_) and sodium acetate buffer (pH_4.5_), and incubated for 15 min. Then, the absorbance was read at 520 and 700 nm and calculated using the following equation: Absorbance = (A_520_ − A_700_)_pH1.0_ − (A_520_ − A_700_)_pH4.5_. Anthocyanin content was calculated based on a cyanidin-3-galactoside equivalent using the equation: Anthocyanin = (Absorbance × MW × 1000)/(ε × C), where a molar absorptivity (ε) is 26,900 and molecular weight (MW) of 449.2 g/mol, and C is the concentration of buffer.

Carotenoid content was determined using high-performance liquid chromatography (HPLC) method [46]. The samples were suspended in a solution containing 3% pyrogallol and 60% KOH, and incubated at 70 °C for 15 min. Next, the sample was extracted with a solution containing 1% NaCl and a mixture of ethyl acetate and hexane. Then, the mixture was centrifuged, and the supernatant was collected. The extraction was repeated until the supernatant became clear, followed by concentration with nitrogen gas and dissolution in ethanol. For measurement of carotenoid content, the sample (10 μL) was injected into a HPLC system comprising with a Capcell Pak UG 120 column (Osaka Soda Co., Osaka, Japan). Carotenoid content was determined at 450 nm, and the carotenoids were separated using a mobile phase of methanol, acetonitrile, and dichloromethane at a flow rate of 1 mL/min. The detected carotenoid concentrations were quantified using a β-carotene equivalent per gram of flesh tissue.

### 4.6. RNA Extraction and qRT-PCR Analysis

RNA extraction and gene expression analysis were performed as described previously (Do et al., 2024 [49]). Briefly, total RNA was extracted using the CTAB method [52], and the quality of the isolated RNA was determined using a spectrophotometer and 1.0% agarose gel. RNA was synthesized into cDNA using a PrimeScript^TM^ 1st strand cDNA synthesis kit (Takara Bio Inc., Shiga, Japan). The expression levels of the candidate genes were analyzed using a qRT-PCR system (LightCycler 480 II; Roche Diagnostics, Mannheim, Germany) and normalized against the *MdActin* reference gene. Primers were obtained from previous studies [22,49], and primer information is provided in Tables S1–S3.

### 4.7. Statistical Analysis

All data were subjected to analysis of variance, and the means among treatments were compared based on Tukey’s HSD test at a significance level of *p* < 0.05 using SPSS software (Version 26; SPSS Inc., Chicago, IL, USA). Principle component analysis (PCA) scores and loading plots were constructed using MetaboAnalyst 6.0 [53].

## 5. Conclusions

The timing of fruit bag removal significantly influences apple quality and coloration. Bag removal close to harvest time noticeably reduced fruit size and SSC. Additionally, fruits subjected to late bag removal, particularly in the 10 DBH group, exhibited lower accumulation of anthocyanin and carotenoid pigments, along with reduced expression of related biosynthetic genes, while chlorophyll content remained higher. However, the late removal of fruit bags at 20 and 10 DBH remarkably reduced the risks of cracking, sunburn, and pathogen infection. Non-bagging and bag removal at 30 DBH posed a high risk of physiological disorders and disease infection, although they improved apple fruit size, quality, and coloration. Therefore, this study suggests that the bag removal timing for ‘Arisoo’ apples should be carefully considered to achieve optimum fruit quality and marketability. Future studies should investigate changes in fruit nutrients and metabolites at different bag removal times.

## Figures and Tables

**Table 1:** Fruit weight and size of ‘Arisoo’ apples treated with no bagging (control) and bag removal at 30, 20, and 10 days before harvest (DBH), as observed at harvest.

Treatments	Fruit Weight (g)	Fruit Size (mm)		
		Length (L)	Diameter (D)	L/D Ratio
No bagging (control)	274.17 ± 3.93 ^z^ a ^y^	79.18 ± 1.68 a	86.72 ± 1.27 a	0.91 ± 0.01 a
Bag removal at 30 DBH	260.68 ± 8.40 a	77.35 ± 2.58 ab	84.90 ± 1.26 ab	0.90 ± 0.01 a
Bag removal at 20 DBH	243.50 ± 5.32 b	75.50 ± 2.00 ab	83.69 ± 1.40 b	0.91 ± 0.01 a
Bag removal at 10 DBH	238.08 ± 5.43 b	74.92 ± 1.89 b	82.04 ± 1.09 b	0.90 ± 0.01 a

^z^ Data indicate mean ± standard error. ^y^ Lowercase letters indicate significant differences among treatments based on Tukey’s HSD test (*p* < 0.05).

**Table 2:** Flesh firmness, soluble solids content (SSC), titratable acidity (TA), SSC/TA ratio, starch pattern index (SPI), and fruit skin color values of ‘Arisoo’ apples treated with no bagging (control) and bag removal at 30, 20, and 10 days before harvest (DBH), as observed at harvest.

Treatments	Flesh Firmness (N)	SSC (Brix)	TA (%)	SSC/TA Ratio	SPI (1–8)	Fruit Skin Color		
						L*	*C*	*h* ^o^
No bagging (control)	58.90 ± 1.24 ^z^ b ^y^	12.85 ± 0.14 a	0.36 ± 0.01 a	35.38 ± 1.20 a	7.87 ± 0.17 a	57.02 ± 0.81 b	28.10 ± 0.86 b	42.77 ± 0.48 c
Bag removal at 30 DBH	60.35 ± 1.45 ab	12.54 ± 0.17 ab	0.38 ± 0.02 a	33.97 ± 1.59 ab	7.80 ± 0.09 a	57.71 ± 1.33 b	28.96 ± 0.90 b	48.72 ± 0.70 b
Bag removal at 20 DBH	60.60 ± 0.75 ab	12.47 ± 0.25 ab	0.36 ± 0.01 a	34.27 ± 0.79 ab	7.88 ± 0.14 a	59.39 ± 0.84 ab	29.99 ± 0.98 b	50.58 ± 0.98 b
Bag removal at 10 DBH	62.25 ± 0.93 a	12.29 ± 0.10 b	0.40 ± 0.02 a	30.74 ± 1.35 b	7.50 ± 0.15 a	61.28 ± 1.48 a	53.52 ± 1.82 a	71.83 ± 1.35 a

^z^ Data indicate mean ± standard error. ^y^ Lowercase letters indicate significant differences among treatments based on Tukey’s HSD test (*p* < 0.05).

## Data Availability

The data is contained within the articles or Supplementary Article.

## Supplementary Materials

The following supporting information can be downloaded at https://www.mdpi.com/article/10.3390/plants14182923/s1: Figure S1: The fruit surface temperature of non-bagged and bagged ‘Arisoo’ apples; Table S1: Primer sequences for chlorophyll degradation-associated genes used in this study; Table S2: Primer sequences for anthocyanin-associated genes used in this study; Table S3: Primer sequences for carotenoid-associated genes used in this study.
