# Supplementary material for: Fruit Bag Removal Timing Influences Fruit Coloration, Quality, and Physiological Disorders in ‘Arisoo’ Apples
Source: Plants (Basel). 2025 Sep 20;14(18):2923. doi: 10.3390/plants14182923 (PMC12473397; doi:10.3390/plants14182923)
Supplement: Supplementary file 1 [file plants-14-02923-s001.zip › plants-3858872-supplementary.pdf]

Supplementary Materials

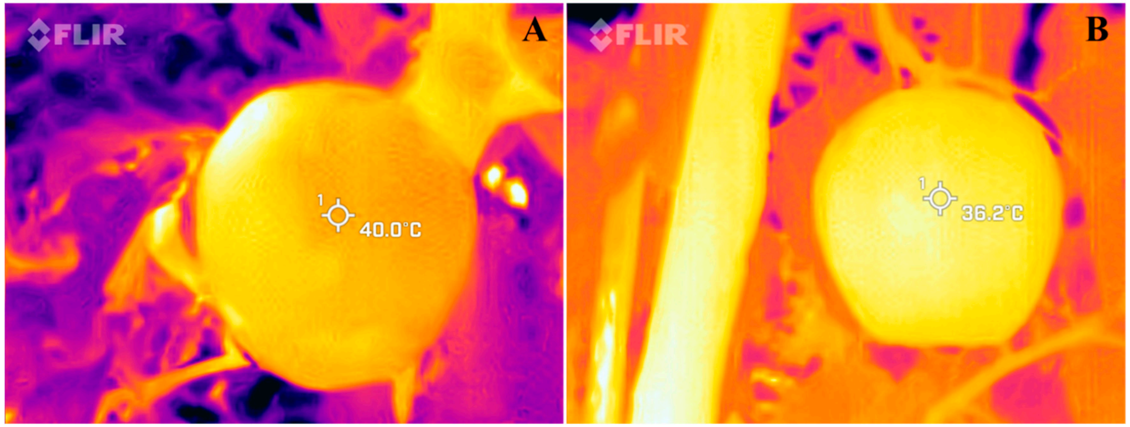

Figure S1. The fruit surface temperature of non-bagged (A) and bagged (B) 'Arisoo' apples. The samples were taken at midday on a sunny day (1 August 2024).

Table S1. Primer sequences for chlorophyll degradation-associated genes.

Name	Gene ID	Forward primer (5'-3')	Reverse primer (5'-3')
MdNYC1	XM_029109831.2	GATCTGTCAATCTGCTGCTGTTC	GCTCATTCAAATCAGCGTCTCC
MdNYC3	XM_008339161.4	GTCTGTCAATCTGCTGCCGT	CATTCAAATCACTGTCTCCACCTG
MdNOL2	XM_029094987.2	CAAGGTTTGCAGCATACGGG	TGCTTCGTAGTTGCACCAGA
MdHCAR	XM_008362212.4	ACTCAAAGTCGGTGAAGCTG	TGTCACACAACCCGCAAC
MdNYE1a	XM_029101034.2	CACCAGGAAAACAACAGCGG	CTGAAAGCTCTGGTGGGTC
MdPPH1	XM_070824389.1	GACCCTTGCTATGTGACCCC	CGTTATCAGCTTCCCTCGCT
MdPAO6	XM_008367917.4	AAGTACGTTCAGAGGCCG	CTGCTTATTCTGCGCCTTC
MdRCCR2	XM_029094039.2	TTTGAAGCGGACGATCATGG	TTACCAGCTTCAATCCCTCCTC
Mdactin	XM_070819956.1	TGACCGAATGAGCAAGGAAATTACT	TACTCAGCTTTGGCAATCCACATC

Table S2. Primer sequences for anthocyanin biosynthesis genes.

Name	Gene ID	Forward primer (5'-3')	Reverse primer (5'-3')
MdPAL	AF494403	TTCCTTGGCAACCCTGTCAC	ATGTCGACCGCCTCAAAGG
MdCHS	AB074485.1	GGACTGGAACTCACTCTTC	GCCGTAATCTGACAACAC
MdCHI	XM_029100826.1	AGGATCACTAACGATCAGCTTCTCT	TGGCCAAACTTTGCTTTGCTG
MdF3H	AB074486	CCAAGCAGTGGTGAACTC	CTTCTCTCCCTCCCTCAC
MdDFR	AF117268	AAGCCTCATCACTGGACT	ATGTGAGAAAGGCAGAGG
MdANS	AB074487	GCTGGAGAAAGAAGTTGG	GGAGGATGAAGGTGAGTG
MdUFGT	AF117267	CAACATCCAAGGTCTCTC	GTCCCATCTGAAGTAGCA
MdMYB1	DQ886414	AGACCAATGTGATAAGACCTCAG	AACCAAAAACTTGTGAAGAGTTC
MdMYB10	XM_029107483.1	TCATCTCTCTACTGCAGTGCCTA	CCAGAAAGACACCTTAGACCTTCG
MdMYB110a	JN711473.1	CTTGAGTGTGATGAGAAAAGGTGC	CCTGTTTAAGCCTGCTTTGTATGG

Table S3. Primer sequences for carotenoid biosynthesis genes.

Name	Gene ID	Forward primer (5'-3')	Reverse primer (5'-3')
MdGGPPS	XM_008356445.3	CCGTGACCCGATTACGATCC	ATGGATTCCGACCCACCAAC
MdPSY	NM_001294092.1	GGGGATATGCCCAGAATCAAGC	CTCTTCCTCTCCTAGCATCTTCTCC
MdPDS	XM_008356843.3	ACATTCCGGTTTCTGACCCG	ACCAAAGCCTTATCGGGTGG
MdZISO	XM_008339306.3	AGCCCTGAGGTTGTGATGTTG	GTCCCCGCAAACAAAACACG
MdZDS	XM_029100719.1	TTTGCCACTAAGACCGAGGC	CTGAGATGAAACCTGCCCCC
MdCRTISO	NM_001329029.1	CCAAAGACACACAGGCGGTA	AGACCATCTATGGCGGTGGT
MdLCYε	XM_008389970.3	CTGGCCGCAAGAAAGGAAAC	CCACATCCAGGTGGGTAAGC
MdLCYβ	XM_008394165.3	ACCCACATGAGCTTGTCGTC	GACACAACAAGTTCCGAAGAGC
MdCRHβ	XM_029106190.1	CGGTGTTTGGGATGGCGTAT	CTTCGAGTTCCTGATGAGCCG
MdZEP	XM_029099177.1	AGGGCCTTCCCCTCACTATC	GCTGTAACCTTGCTTGGGTCT
